# Potential Mechanisms of Partial/Transient Response or Resistance to Daratumumab Therapy: A Focus on Anti-Daratumumab Antibodies and Urinary Daratumumab Loss

**DOI:** 10.3390/antib15040057

**Published:** 2026-07-03

**Authors:** Marco Allinovi, Luca Malatesta, Tiziana Biagioli, Elisabetta Antonioli, Federico Perfetto

**Affiliations:** 1 Nephrology, Dialysis and Transplantation Unit, Careggi University Hospital, 50134 Florence, Italy; luca.malatesta@unifi.it; 2 Tuscan Regional Amyloidosis Centre, Careggi University Hospital, 50134 Florence, Italy; federico.perfetto@unifi.it; 3 General Laboratory, Department of Diagnostics, Careggi University Hospital, 50134 Florence, Italy; biagiolit@aou-careggi.toscana.it; 4 Haematology Unit, Careggi University Hospital, 50134 Florence, Italy; antoniolie@aou-careggi.toscana.it

**Keywords:** daratumumab, anti-drug antibodies, anti-daratumumab antibodies, daratumumab resistance, multiple myeloma, amyloidosis

## Abstract

Daratumumab, a human IgG1 monoclonal antibody targeting CD38, is widely used in multiple myeloma and AL amyloidosis. Despite its clinical success, many patients fail to achieve durable responses or relapse, underscoring the importance of understanding resistance mechanisms. Drawing on experience from other better-studied monoclonal antibodies, resistance to daratumumab can be categorized into four main mechanisms: (1) reduced CD38 expression on plasma cells; (2) increased expression of complement inhibitory proteins (CD55/CD59), impairing complement-mediated cytotoxicity; (3) reduced drug bioavailability due to urinary loss in non-selective nephrotic syndrome; and (4) the development of neutralizing anti-daratumumab antibodies. Anti-drug antibodies (ADAs) may represent a potential mechanism of treatment failure through effects on pharmacokinetics, efficacy, and safety, even in patients on daratumumab therapy. Seven different trials have tested anti-daratumumab antibodies. Among them, anti-daratumumab antibodies were identified in only 0–2.4% of patients, and only in a small portion of these has it been proven to be neutralizing. Overall, ADAs appear rare, but these findings are likely underestimated due to short follow-up and suboptimal timing of assessment. In conclusion, standardized ADA monitoring, particularly months after treatment interruption or in cases of inadequate response or infusion-related reactions, may improve patient management and therapeutic outcomes.

## 1. Introduction

Monoclonal antibodies have revolutionized modern therapy by offering targeted treatments with greater efficacy and fewer side effects than traditional therapies [1]. However, despite the enormous innovation they have represented and continue to represent, their effectiveness shows considerable variability in clinical practice. Among the most studied causal mechanisms of unsatisfactory response (partial/transient response or resistance) is certainly the development of anti-drug antibodies (ADAs) [2].

**Figure 1:**
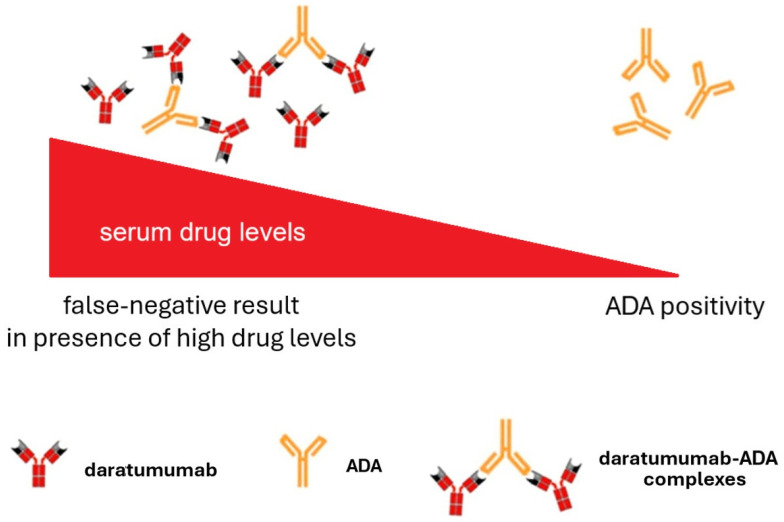
Daratumumab available in circulation binds to anti-drug antibodies (ADAs), and these antibody–drug conjugates are no longer detectable/quantifiable by the ELISA kit (false negativity). Consequently, testing during therapy may produce false negatives due to interference with the serum-free daratumumab. The more months that pass since the last drug administration, the lower the concentration of free drug in circulation, and the more likely it is to identify the presence of ADAs (if present).

Indeed, all therapeutic agents exhibit some degree of immunogenic potential. Therapeutic monoclonal antibodies are no exception, as their administration may elicit ADAs, which can modify pharmacokinetic and pharmacodynamic profiles [3,4]. It has been demonstrated that ADAs are generally detected several (from 3 to 12) months after the last monoclonal antibody infusion, probably because the drug available in circulation binds to ADAs (antibody–drug conjugate; neutralizing activity) and these are no longer detectable/quantifiable (false negativity) (Figure 1) [5].

ADAs, when neutralizing, reduce serum drug concentrations, are associated with infusion-related reactions, significantly reduce drug efficacy, and are associated with more frequent and earlier relapses of the underlying disease [6,7].

The immunogenicity of therapeutic monoclonal antibodies and the development of ADAs are influenced by treatment-related factors such as dosing regimen, route of administration, and the carrying out of multiple previous therapies, which in a certain sense “immunize” the patient [8,9].

In addition, the composition of the drug itself plays a crucial role in the development of ADAs. Antibodies of nonhuman origin (usually murine or chimeric) are frequently immunogenic when administered to human patients. This is why the introduction of humanization method has substantially decreased the risk of immunogenicity from nonhuman antibodies, and it is for the same reason that more fully human or humanized ones are being developed [10,11]. Among these, there is certainly daratumumab, a human IgG1 monoclonal antibody that targets CD38, a surface protein highly expressed on neoplastic plasma cells, which therefore finds its strong application in some types of hematological diseases, such as multiple myeloma and AL amyloidosis [12,13,14].

Although daratumumab has demonstrated impressive efficacy, a substantial proportion (up to one third) of patients with multiple myeloma or AL amyloidosis treated by a daratumumab-based regimen fail to achieve deep and durable hematological remissions and most eventually relapse, associated with poor outcomes [15,16].

However, if we wanted to exemplify it in different points, the development of ADAs could be only one of the several potential mechanisms listed below that could reduce daratumumab efficacy, which we could group into four categories [17]: reduction/downregulation of the CD38 expression, increased level of the complement inhibitory proteins, reduced bioavailability due to urinary daratumumab loss in patients with non-selective nephrotic-range proteinuria, and the occurrence of anti-daratumumab antibodies.

## 2. Reduction or Downregulation of the CD38 Expression on the Myeloma Cells

Daratumumab acts by binding to the cell surface via CD38, meaning that its efficacy depends directly on the density of CD38 on the surface of plasma cells: the more CD38 there is, the more vulnerable the cell is to the cytotoxic mechanisms of daratumumab. Among the mechanisms of drug resistance is therefore reduction or downregulation of CD38 expression on the plasma cell membrane, which can occur through various biological mechanisms, including (a) internalization of the bound antibody–antigen complex and therefore reduction of the amount of CD38 on the surface; (b) “shedding,” the mechanism by which some cells release CD38 into the microenvironment, reducing its expression on the membrane; (c) transcriptional regulation, by which malignant cells can decrease transcription of the CD38 gene; or finally, (d) clonal selection, by which cells with naturally lower CD38 expression survive treatment and become dominant [18]. Some recurrent cytogenetic abnormalities, such as the acquisition or amplification of chromosome 1q21, have been associated with a lower response to daratumumab through this type of resistance, whereby 1q21+ myeloma cells would show a lower expression of CD38 mediated by an increase in IL-6R/JAK/STAT signaling [19,20]. Precisely because of these resistance mechanisms just listed, preclinical studies in recent years have shown that agents including lenalidomide, panobinostat, all-trans retinoic acid, and DNA methyltransferase inhibitors can upregulate CD38 expression, potentially enhancing the efficacy of anti-CD38 therapies [21].

## 3. Increased Level of the Complement Inhibitory Proteins

Daratumumab acts by potently inducing complement-dependent cytotoxicity (CDC) in cell lines. However, the intensity of lysis is influenced by the expression of complement inhibitory proteins (CIPs) such as CD55 and CD59, which limit the formation of the membrane attack complex (MAC). Cells with higher CIP expression are therefore less sensitive to CDC, as complement inhibition drastically reduces cytotoxic efficacy [22]. As demonstrated in the study by Nijhof et al. [23] on patients with relapsed/refractory multiple myeloma (RRMM), a significant reduction in CD38 was observed at disease relapse, but also an increase in CD55 and CD59. The upregulation of CIPs correlated with reduced sensitivity to ex vivo CDC. Furthermore, there was no strong association between baseline CIPs and initial response, suggesting an acquired mechanism to therapy, as subsequently confirmed by Iversen et al., who reinforced the concept of “tumor adaptation” by proposing dynamic monitoring of CIPs [24].

Future combined strategies could restore MAC formation in these settings, as demonstrated by Wang H. et al., in whose study they evaluated direct blockade of CD46 and CD59 using Ad35K++ (CD46 inhibitor) and rILYd4 (CD59 inhibitor) and demonstrated that inhibition of CIPs significantly increased daratumumab-induced CDC [25].

## 4. Urinary Drug Loss

Daratumumab, as an IgG1κ, exhibits clearance predominantly mediated by intracellular catabolic processes after endocytosis and by target-mediated mechanisms, similar to other monoclonal antibodies. The high molecular weight (148 kDa) prevents significant glomerular filtration, making renal elimination clinically negligible. Therefore, under physiological conditions, urinary clearance does not represent a significant route of drug elimination [26].

However, one of the major mechanisms of loss of efficacy of therapeutic monoclonal antibodies is their loss in the urine and therefore the failure to reach therapeutic serum concentrations, especially in nephropathies characterized by non-selective nephrotic syndrome. In fact, in patients with severe proteinuria and persistent kidney disease, relatively big quantities of high-molecular-weight proteins (such as IgG and consequently therapeutic monoclonal antibodies as well) pass into the urine as an expression of severe podocyte damage. The studies by Jacobs et al. [27] and Allinovi et al. [28] demonstrated how renal integrity can influence the pharmacokinetics of monoclonal antibodies, identifying an important urinary rituximab loss in patients with glomerular damage, leading to lower serum drug concentrations in patients with non-selective nephrotic syndrome.

Although less extensively studied, other monoclonal drugs have also been demonstrated in the urine of patients with severe proteinuria. In particular, one study documented that up to 13% of the adalimumab dose was lost in the urine of patients with focal segmental glomerulosclerosis (FSGS), a glomerulonephritis characterized by nephrotic syndrome [29]. In another study, a kidney transplant recipient with chronic antibody-mediated rejection had detectable tocilizumab in urine and also presented the highest urine albumin-to-creatinine ratio in the study population (ACR = 948 mg/mmol) [30].

Although it has been studied on other types of monoclonal antibodies (such as rituximab, adalimumab, and tocilizumab), it is rational to think that this mechanism of drug resistance due to urinary drug loss could be applicable to all types of monoclonal antibodies and therefore also to daratumumab, in particular those patients with non-selective nephrotic syndrome, which is frequently present in AL amyloidosis or other MGRS. However, although in Kimmich’s work the presence of daratumumab was observed as a compatible band on electrophoresis in patients with nephrotic proteinuria, no quantitative measurement was performed, nor was a study carried out to calculate concentrations, urinary clearance, or systemic impact [31]. Only one study showed that a higher proteinuria was associated with earlier disappearance of daratumumab (OR 2.79), suggesting a possible urinary drug loss [32].

Further studies are urgently needed to confirm the applicability of these concepts to daratumumab.

## 5. Anti-Drug Antibodies (ADAs)

Studies on ADAs are growing in the literature, confirming their role in reducing or losing pharmacological efficacy [5]. The mechanisms of immunogenicity are heterogeneous. For example, some pharmacological and immunological studies suggest that intermittent or low exposure dosing regimens may be associated with increased ADA incidence, while other studies suggest sustained exposure through higher or more frequent dosing might promote immune tolerance and reduce ADA formation. Furthermore, it seems that subcutaneous administration is generally more immunogenic than the intravenous route, probably due to prolonged exposure to antigen-presenting cells in peripheral tissues [8,9].

Among these studies, given its heterogeneous therapeutic applications, the greatest attention has been attracted by resistance mechanism studies focused on anti-rituximab antibodies, in which the presence of anti-rituximab antibodies was identified in 20–50% of treated patients, correlated with lower serum rituximab levels, incomplete B cell depletion, in vitro neutralization, lower clinical remission, and higher rates of clinical relapse [6,11,33]. Furthermore, the prevalence of ADAs would seem to vary depending on the underlying pathology and reached a higher prevalence in autoimmune diseases compared with hematological disorders, likely due to the intrinsic autoimmune nature of the underlying disease [34,35]. In addition to evaluating its incidence, obviously, its impact on clinical practice has been evaluated, showing how the presence of serum ADA is associated with a lower depletion [33] or a more rapid repopulation of B cells [36], with a possible impact on the efficacy of the treatment, and how the neutralizing effect on B cells could be confirmed in vivo [37].

We conducted a comprehensive literature search across the PubMed, Google Scholar, Web of Science, and Cochrane Library databases from their inception to 5 June 2026. The search strategy was designed using a combination of Medical Subject Headings (MeSHs) and free-text terms to identify relevant studies focused on daratumumab immunogenicity. Specifically, the search string integrated keywords related to the drug (“daratumumab”, “dara”, or “Darzalex”), terms capturing anti-drug antibody responses (“anti-drug antibodies”, “anti-daratumumab antibodies”, “ADA”, “immunogenicity”, or “neutralizing antibodies”), and descriptors for the target hematologic conditions (“multiple myeloma”, “plasma cell dyscrasia”, or “hematologic malignancies”).

We reviewed all articles written in English, and we selected those that provided information on detection of anti-daratumumab antibodies. The exclusion criteria were as follows: expert opinions, ongoing studies, reviews, articles not written in English, articles that do not mention ADAs, and studies on animal models. Two investigators (L.M. and M.A.) independently screened all identified papers for inclusion based on the study title and abstract. A total of seven published studies were included. We collected information on timing of development, antibody titer, incidence, and neutralizing effect.

This review summarizes the current evidence on the different mechanisms of resistance to daratumumab, the prevalence of anti-daratumumab antibodies, and the clinical significance and underlying mechanisms of anti-drug antibodies development, highlighting key aspects that have emerged from the most recent studies.

Despite many articles on ADA against various monoclonal antibodies [38] and despite growing evidence, ADAs are not routinely tested in daily clinical practice, with their analysis generally being placed in early stages of drug development. Measurement of ADA to assess the incidence of ADA in a clinical trial is a critical step in immunogenicity assessment while creating a protein-based therapy. ADAs detection can involve important implications for treatment strategies of cancer patients, guiding therapeutic adjustment [39].

Starting from the studies on atezolizumab, we can gain a point of view that can be applied to the experience with daratumumab. In fact, a recent interesting study [40] has revealed that the methods commonly employed throughout the industry to analyze ADA occurrence offer only a narrow perspective on immunogenicity in cancer trials, where identifying ADA can be obscured by both the administered drug dose and the loss of patients from the study. The same research also demonstrated that relying on infrequent sample collection and/or utilizing ADA assays lacking adequate drug interference resistance can lead to a considerable underestimation of ADA incidence.

In confirmation of this, a low incidence of ADA has been reported (<3%) in initial studies and trials on pembrolizumab [41], ipilimumab [41], and rituximab [42,43], while recent studies documented a higher incidence of ADA (>25%) for pembrolizumab [44], ipilimumab [45], and rituximab [5,46] in some clinical settings.

Moreover, it is commonly believed that human or humanized monoclonal antibodies are less immunogenic than murine or chimeric antibodies, suggesting that the search for anti-daratumumab antibodies is intended to identify small numbers. However, in recent studies, ADAs have been found in 30–50% of patients treated by adalimumab (human), [47,48] obinutuzumab (humanized) [49], and pembrolizumab (humanized) [44].

We identified seven studies that investigated the presence of anti-daratumumab antibodies in patients undergoing daratumumab-based regimens [50,51,52,53,54,55,56].

First, a phase 1/2 open-label trial in patients with multiple myeloma treated by daratumumab/lenalidomide/dexamethasone showed that no anti-daratumumab antibodies were detected during the course of the study in two different cohorts of 13 and 24 evaluable patients, respectively. However, since the therapeutic protocol is characterized by consecutive cycles with frequent intravenous daratumumab infusions and a short follow-up, the data on anti-daratumumab antibodies are not reliable as they are burdened by a very high risk of false negatives [50].

Second, in a phase 1b open-label trial in patients naive to anti-CD38 therapy with multiple myeloma who received ≥1 dose of subcutaneous daratumumab, the incidence of anti-daratumumab antibodies was assessed. Although it is not specified in how many patients ADA was tested, anti-daratumumab antibodies were detected in one patient, appearing as neutralizing, transient, and without affecting pharmacokinetic properties. Even in this case, there is a high risk of false negatives in relation to consecutive cycles with frequent subcutaneous daratumumab infusions and a short follow-up [51].

Third, the final analysis of a phase 3 non-inferiority COLUMBA study, comparing the subcutaneous (SC) formulation with the intravenous (IV) formulation in patients with relapsed or refractory multiple myeloma, provided the most data and the longest follow-up. With extended follow-up, the incidence of treatment-emergent anti-daratumumab antibodies was 0.4% of patients in the SC group (1/260) and 2.6% in the IV group (6/258). The one patient in the DARA SC group who tested positive for anti-daratumumab antibodies also tested positive for neutralizing antibodies, and five of six patients in the DARA IV group who tested positive for anti-daratumumab antibodies also tested positive for neutralizing antibodies. However, although prolonged follow-up reduces the temporal bias in the ability to develop antibodies, the residual risk of underestimation of anti-daratumumab antibodies (false negatives) remains partially due to the high and constant serum concentration of daratumumab circulating during continuous therapy, which can saturate the binding sites of immunological tests to the same antibodies (drug interference) [52].

Fourth, the phase 2 PLEIADES study evaluated the immunogenicity of subcutaneous daratumumab when administered not as a monotherapy, but in combination with standard treatment regimens (such as DvRd, DVMP, and DRd) in various lines of treatment for multiple myeloma. Even in this multidrug setting, the incidence of treatment-emergent anti-daratumumab antibodies was absent, with none being detected in any patient analyzed. However, 5.9% of antibodies was reported to be anti-rHuPH20, the recombinant human hyaluronidase excipient used for subcutaneous injection, but none of these antibodies were neutralizing [53].

Fifth, the final analysis of the phase 3 OCTANS study extended the immunological safety assessment to the Asian population with newly diagnosed multiple myeloma who were ineligible for transplant, treated with daratumumab in combination with bortezomib, melphalan, and prednisone (D-VMP). With robust follow-up exceeding 3 years, the study documented a low immunogenicity rate, identifying only one patient positive for anti-daratumumab antibodies out of a total of 130 evaluable subjects (approximately 0.8%) [54].

Sixth, a randomized, double-blind, phase 1 study evaluated the immunogenicity of the potential biosimilar of daratumumab QL2109 compared with the reference subcutaneous daratumumab in 222 healthy volunteers. The incidence of anti-daratumumab antibodies was closely comparable and overlapping between the two groups (110 subjects treated with QL2109 and 112 with the original drug). Furthermore, neutralizing antibodies were not detected in any participant, confirming that the biosimilar exhibits the same immunological safety as the original drug, even in healthy subjects [55].

Finally, the phase 2 study conducted by Huang et al. evaluated daratumumab monotherapy in relapsed or refractory nasal-type natural killer/T-cell lymphoma (NKTL). Even in this patient cohort, characterized by a tumor microenvironment completely different from that of myeloma, 0% of evaluable patients developed treatment-emergent anti-daratumumab antibodies [56].

Among the seven identified trials (Table 1), anti-daratumumab antibodies were identified in only 0–2.4% of patients, and only in a small portion of these have they proven to be neutralizing.

However, interpretation of data on the prevalence of anti-daratumumab antibodies and their potential clinical impact is limited by variability in the timing of anti-daratumumab antibody measurements.

**Figure 2:**
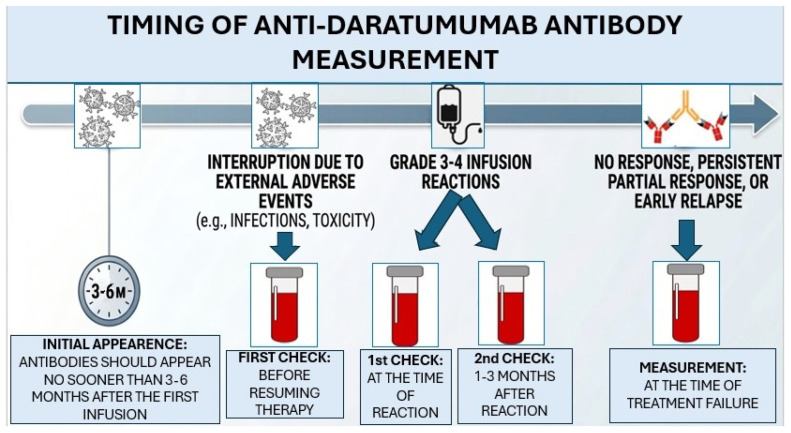
In relation to previously published articles on different therapeutic monoclonal antibodies and on previously published pharmacokinetic studies of intravenous and subcutaneous daratumumab, we propose standardizing the monitoring of anti-daratumumab antibodies, at least after 3 to 6 months following last infusion, in particular in those cases characterized by (1) grade 3–4 infusion reactions, (2) before daratumumab readministration in those patients who had temporarily stopped taking the drug, (3) in those patients who have not achieved satisfactory hematological remission (e.g., VGPR or complete remission), (4) or in those patients who experienced an early relapse.

Because anti-daratumumab antibodies may decrease the efficacy of daratumumab-based regimens, leading to partial, delayed or transient response, or even resistance, it would therefore be appropriate to standardize the assessment of anti-daratumumab antibodies, which should be routinely monitored in patients previously treated with daratumumab in patients with unsatisfactory hematological remission or if re-treatment is being considered (e.g., in case of relapse). The neutralizing effect of anti-daratumumab antibodies, which theoretically could lead to a significant reduction in the effective serum concentration of daratumumab, could be masked in most cases by therapeutic regimens characterized by repeated daratumumab infusions (Figure 2).

High and/or repeated doses of therapeutic monoclonal antibodies can induce immune tolerance and reduce ADA formation. Conversely, low residual drug levels have been associated with increased immunogenicity. Nonetheless, this relationship may vary depending on the disease and therapeutic context. Moreover, development of ADAs is generally reduced by concomitant immunosuppressive treatment, while it is increased by longer intervals between drug administrations; thus, regular daratumumab infusion regimens with concomitant chemotherapy agents should be preferred [57].

Infusion-related reactions in ADA-positive patients have been widely documented and include a broad spectrum of clinical manifestations (such as anaphylaxis, fever, headache, urticaria, and rashes) [58,59]. For this reason, another optimal time to test for the presence of anti-daratumumab antibodies is in case of infusion-related reactions and in the following days/weeks.

Enzyme-linked immunosorbent assay (ELISA) kits are commercially available for the detection of anti-daratumumab antibodies. These tests identify ADAs in patient serum and are commonly used to assess immunogenicity [60].

The timing of testing for anti-daratumumab antibodies in the seven reported studies is unknown, but it is clear that in a protocol involving monthly drug administration, it is highly unlikely to identify ADAs at a random time point.

Taking inspiration from a different model, serial monitoring of anti-rituximab antibody development in rituximab-naive patients has allowed for the demonstration that ADAs are identifiable at least 3 months after the last rituximab administration [5].

Considering the pharmacokinetic studies of intravenous and subcutaneous daratumumab [26,32,61,62,63], and considering that anti-daratumumab antibodies cannot be detected if an identifiable concentration of daratumumab is present in circulation (false negative result), anti-daratumumab antibodies might appear 3 to 6 months after cessation of daratumumab infusion, or even earlier in presence of proteinuria [32], hypoalbuminemia, and IgG type myeloma (vs. non-IgG) [62], which were identified as variables able to influence daratumumab pharmacokinetics by linearly reducing the drug concentration.

It is therefore advisable that anti-daratumumab antibodies should be tested at least 2–3 months after the last administration, in case of a transitory interruption or at the end of the monthly daratumumab administration protocol.

In conclusion, we believe that research is progressing rapidly in the study of anti-drug resistance, particularly to ADAs, but we are still far from fully understanding the mechanisms of resistance focused on anti-daratumumab antibodies, a drug with growing current use and future prospects. Although the reported rate of anti-daratumumab antibodies is currently low, this could be much higher in studies in the coming years, as has happened with many other monoclonal antibodies. We recommend monitoring anti-daratumumab antibodies, also in light of experience with other ADAs, several months after the last administration, and especially in the event of adverse reactions to infusion, in patients with partial or delayed response to therapy; in patients with no response to therapy; and in patients who, for various reasons, have received therapy intermittently.

## Figures and Tables

**Table 1:** Main data relative to the seven studies that investigated the presence of anti-daratumumab antibodies.

Study	Treatment	N°	Response (ORR/NRs)	ADAs (Pos/Neg)
GEN503 (2019) [50] Daratumumab Combination Therapy IV	Total	45		
	Variable dose	13	11 (84.6%)/2	0/13
	Standard dose	32	26 (81.3%)/6	0/24
PAVO (2019) [51] Daratumumab Monotherapy SC	Total	53		
	1200 mg	8	2 (25.0%)/8	0/NA
	1800 mg	45	19 (42.2%)/45	1/NA
COLUMBA (2022) [52] Daratumumab Monotherapy	Total	522		
	SC	263	115 (43.7%)/148	1/259
	IV	259	103 (39.8%)/156	6/252
PLEIADES (2021) [53] Daratumumab Combination Therapy SC	Total	199		0/199 5.9% antibodies anti-rHuPH20; none neutralizing
	D-VRd	67	NA	
	D-VMP	67	60 (90%)/7	
	D-Rd	65	61 (93.8%)/4	
OCTANS (2024) [54] Daratumumab Combination Therapy IV	Total	220		
	D-VMP	146	132 (90.4%)/14	1/144
	VMP	74	60 (81.1%)/14	-
Hu X. et al. (2025) [55] Study on Biosimilar (Healthy Volunteers)	Total	222	-	222
	QL2109 (Biosimilar) SC	110	-	ADA positivity unknown, but comparable between the 2 groups
	Daratumumab SC	112	-	0/222 (0%) NAb
Huang et al. (2021) [56] Daratumumab Monotherapy IV	Total	32	8 (25%)/24	0/26

Legend: ORR: Objective response rate (responders); NRs: non-responders; ADAs: anti-drug antibodies; Pos: positive; Neg: negative; SC: subcutaneous; IV: intravenous; NAb: neutralizing anti-drug antibodies; D-VRd: daratumumab plus bortezomib/lenalidomide/dexamethasone; D-VMP: daratumumab plus bortezomib/melphalan/prednisone; D-Rd: daratumumab plus lenalidomide/dexamethasone; VMP: bortezomib/melphalan/prednisone.

## Data Availability

The data underlying this article will be shared on reasonable request made to the corresponding author.
